# Plasma miR-145-5p Levels and Risk of Future Cancer—Results from the HUNT Study

**DOI:** 10.3390/ijms26052191

**Published:** 2025-02-28

**Authors:** Christopher Antoun, Julia Oto, Vânia M. Morelli, Kristian Hveem, Sigrid K. Brækkan, Pilar Medina, John-Bjarne Hansen

**Affiliations:** 1 Thrombosis Research Group (TREC), Department of Clinical Medicine, UiT—The Arctic University of Norway, 9019 Tromsø, Norway; vania.m.morelli@uit.no (V.M.M.); sigrid.brakkan@uit.no (S.K.B.); john-bjarne.hansen@uit.no (J.-B.H.); 2 Haemostasis, Thrombosis, Arteriosclerosis and Vascular Biology Research Group, Health Research Institute Hospital La Fe, 46026 Valencia, Spain; julia_oto@iislafe.es (J.O.); medina_pil@gva.es (P.M.); 3 Thrombosis Research Center (TREC), Division of Internal Medicine, University Hospital of North Norway, 9019 Tromsø, Norway; 4 HUNT Center for Molecular and Clinical Epidemiology, Norwegian University of Science and Technology, 7491 Trondheim, Norway; kristian.hveem@ntnu.no; 5 HUNT Research Center, Department of Public Health and Nursing, Norwegian University of Science and Technology, 7600 Levanger, Norway

**Keywords:** microRNAs, cancer, follow-up studies, biomarkers, cohort studies, proportional hazards models

## Abstract

MicroRNA-145-5p (miR-145) has been reported to regulate multiple oncogenes and is considered a tumor suppressor. However, it remains unknown whether the level of plasma miR-145 can serve as a risk biomarker for future cancer. Using a population-based cohort (*n* = 1740) derived from the Trøndelag Health Study (HUNT), we investigated whether plasma miR-145 levels were associated with (1) first life-time cancer, (2) cancer stage at diagnosis, and (3) 2-year all-cause mortality after cancer diagnosis. Cox regression analysis was used to estimate hazard ratios (HR) and 95% confidence intervals (CI). Our findings showed that individuals in the highest quartile of plasma miR-145 levels had a 44% increased risk of developing cancer compared to those in the lowest quartile, independent of age, sex, body mass index, or smoking status (HR 1.44, 95% CI 1.03–2.00 *p* < 0.05). However, no association was observed between quartiles of miR-145 levels and the risk of being diagnosed with a metastatic cancer, or the risk of 2-year mortality after cancer diagnosis. Our findings suggest that high plasma miR-145 levels are associated with increased cancer risk without affecting the severity of the cancer at diagnosis or affecting the short-term prognosis.

## 1. Introduction

Estimates from the GLOBOCAN study in 2022 indicated that around one in nine men and one in twelve women died from cancer [1]. Timely diagnosis can improve these figures [2], but screening efforts for early detection bring their own set of challenges [3,4]. Hence, identifying novel and minimally invasive biomarkers that improve cancer risk stratification might minimize the personal distress and socioeconomic burden of cancer and cancer management in society.

Despite its heterogenous nature, a phenomenon that is common across virtually all human malignancies is the dysregulation of microRNAs (miRs) [5]. These small (~22 nucleotides) non-coding RNAs inhibit translation of their target messenger RNAs (mRNAs) [6], including those involved in processes promoting tumor initiation and progression [7]. Deviations in these processes, such as insensitivity to anti-growth signals, uncontrollable growth, and evasion of apoptosis, have been deemed as the hallmarks of cancer [8]. Moreover, miR genes are frequently located in so-called fragile chromosomal regions that are often altered in cancer [9]. One such example is the chromosomal region 5q32-33, harboring miR-145, which is repeatedly deleted in blood cancers [10].

The miR-145 gene is conserved and expressed in stromal cells such as fibroblasts which form part of the tumor microenvironment [11,12,13]. Cui et al. [14] summarized key cancer pathways regulated by miR-145, including those involved in cell proliferation, apoptosis and invasion, demonstrating that it is a potent tumor suppressor. Moreover, miR-145 has been shown to take part in the regulatory network of the central tumor suppressor p53, which is induced in response to DNA damage [15]. Even though growing evidence from experimental and observational studies supports a tumor-suppressor role for miR-145, this notion has been disputed. The miR143/145 cluster has also been demonstrated to promote tumorigenesis in a mouse model with lung cancer [16]. The discrepant results might be partially explained by altered mechanisms of action of miRs due to variations in the cellular context over time in normal tissues and the tumor environment [17], which was supported by a review article summarizing both the anti-oncogenic and the pro-oncogenic properties of miR-145 [18]. Consequently, the physiological changes introduced by the onset of cancer make it more challenging to elucidate the role(s) of miR-145 in tumor development and progression. Although several case-control studies have shown downregulation of miR-145 in cancer patients across various sample types [19,20], supporting a tumor-suppressor role for miR-145, it remains unknown whether plasma miR-145 levels are associated with risk of future cancer.

Therefore, we set out to explore the relationship between plasma miR-145-5p (miR-145) levels and risk of future first-lifetime cancer in a cohort derived from the general population. We aimed to investigate whether plasma miR-145 levels were associated with (1) risk of future cancer, (2) cancer stage at diagnosis, and (3) 2-year mortality after cancer diagnosis.

## 2. Results

The distribution of baseline characteristics for all study participants and according to miR-145 quartiles is given in Table 1. The mean age of the population was about 65 years, which slightly increased to 67 years for participants with the highest miR-145 expression levels. Since the quartiles were conceived for each sex separately, the proportion of females remained constant at 55% in each quartile. The mean smoking pack-years decreased from 8.6 pack-years to 6.4 pack-years with increasing quartiles of miR-145. The other baseline characteristics were essentially similar across quartiles of miR-145 levels.

The characteristics of the 300 patients diagnosed with cancer during the follow-up period (max. 13.2 years) are shown in Table 2. On average, there were 6.4 years between enrolment and diagnosis. The mean age at diagnosis was 76 years, with a lower proportion of females (42%) compared to males. Regarding properties of cancers, 35% were initially diagnosed at a local stage, 29% regional, and 19% distant metastases. The most common cancer site was the gastrointestinal tract, accounting for 25% of cases, followed by prostate (19%), lung (9%) and hemo/lymph (9%) cancers. The cancers categorized as “Others” are given in Table S1.

**Figure 1:**
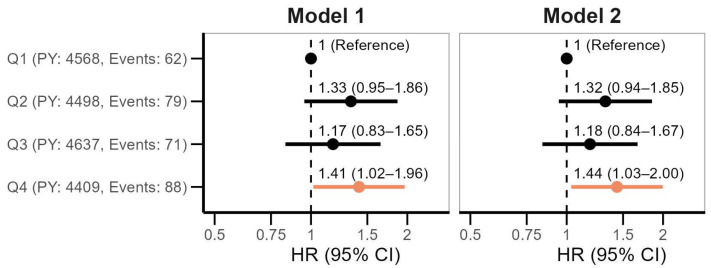
Hazard ratios (HRs) with 95% confidence intervals (CIs) of overall cancer according to quartiles (Q) of plasma miR-145. Model 1: adjusted for age, sex and body mass index; Model 2 adjusted for Model 1 + smoking pack-years. PY refers to person-years.

The hazard ratios (HRs) with 95% confidence interval (CI) of overall cancer across quartiles of miR-145 are shown in Figure 1. Participants in the highest quartile of miR-145 had a 41% increased risk of cancer (HR 1.41, 95% CI 1.02–1.96, *p* < 0.05) compared with those in the lowest quartile of miR-145 in a model adjusted for sex, age and body mass index (BMI) at baseline (Model 1). The risk estimates slightly increased after additional adjustment for pack-years (Model 2) (*p* < 0.05). Notably, the increase in HR did not follow a linear pattern across rising quartiles of miR-145 expression. The association between continuous plasma miR-145, modeled as a spline with four degrees of freedom, and risk of cancer is shown in Figure S1. The fully adjusted model suggested a non-linear pattern of miR-145 on cancer risk. As indicated by the wide confidence intervals, risk estimates at more extreme values were supported by only a few observations.

**Figure 2:**
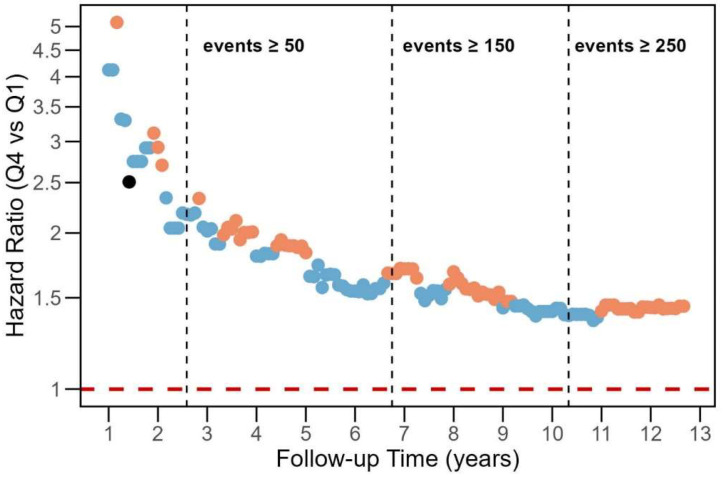
Estimated hazard ratios (HRs) of cancer as a function of follow-up time from blood sampling to cancer event. Analysis was adjusted for sex, age, body mass index and smoking pack-years. Individuals with the miR-145 level in the highest quartile (Q4) were compared with those with miR-145 in the lowest quartile (Q1; reference). Dashed vertical lines indicate when the number of cancer events exceeded the number marked in the plot, and the red dashed horizontal line indicates a HR of 1.00.Blue dots represent HRs with *p*-values < 0.1, orange dots indicate HRs with *p*-values < 0.05, and black dots signify HRs with *p*-values > 0.1.

To assess the potential of regression dilution bias, the HRs for Q4 versus Q1 were estimated with varying follow-up times from 1 year to 13.2 years (Figure 2). The risk was highest during the first two years of follow-up, although the number of cancer events was < 50. After 2.5 years, the HR decreased and continued to decline gradually over time, yet it consistently remained above 1.35. Results from the additional sensitivity analysis that excluded participants who received a cancer diagnosis within one year of enrollment (*n* = 22) are shown in Figure S2. The risk estimates for Model 2 decreased from 1.44 (95% CI 1.03–2.00) to 1.34 (95% CI 0.96–1.89). Figure S3 shows the variation of the mean value of miR-145 by increasing follow-up periods. Among participants followed for up to 4 years, those who developed cancer had higher mean miR-145 levels compared to their sex and age-matched controls, with the greatest difference in the mean trend occurring in the first three years. Interestingly, the smoothing lines intersect after 4 years, indicating a reversal in the pattern. For participants followed for 7 years or more, the mean miR-145 levels were once again higher in those with cancer than those without.

Results from re-calculating risk estimates as a function of increasing pack-years are shown in Figure S4. The risk estimates for Q4 were highest (HR 1.94, 95% CI 1.28–2.96) when including only people with pack-years ≤ 16, and started to decrease after that before stabilizing at around 1.43. In analyses restricted to people with zero pack-years (*n* = 970, events = 171), the HR was 1.62 (95% CI 1.02–2.56).

Risk estimates for each general cancer site are shown in Table S2. Elevated miR-145 levels were particularly associated with high risk of lung cancer (HR 4.24 95% CI: 1.18–15.23), gastrointestinal (GI) cancers, malignant melanomas and cancers defined as “Others” (Table S2), but these subgroup analyses should be interpreted with caution due to the low numbers of events in each subcategory.

**Figure 3:**
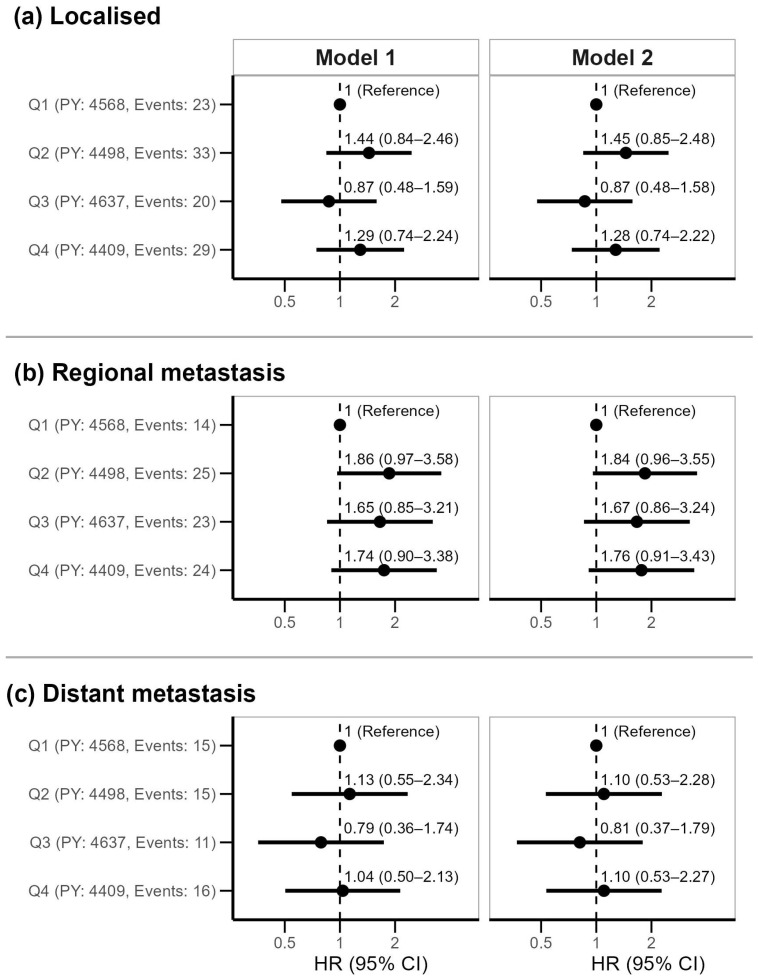
Hazard ratios (HRs) with 95% confidence intervals (CIs) of cancer stage at diagnosis according to quartiles (Q) of plasma miR-145. Model 1: adjusted for age, sex and body mass index at baseline; Model 2 additionally is adjusted for pack years. PY refers to person-years.

The HRs for cancer stages at diagnosis are given in Figure 3. No clear trend was observed for either localized or distant metastasis. For regional metastasis, the HRs displayed a non-linear relationship with quartiles of miR-145, where individuals with miR-145 levels in the lowest quartile had a lower risk of regional metastasis compared to those with higher miR-145 levels.

**Figure 4:**
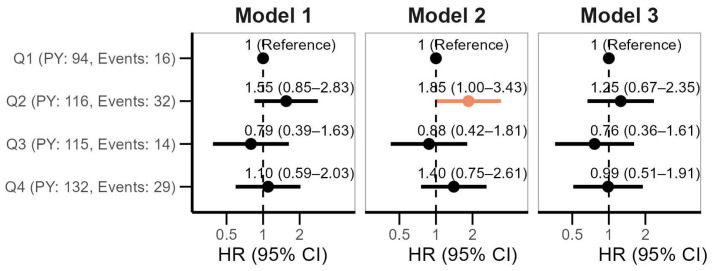
Hazard ratios (HRs) with 95% confidence intervals (CIs) of 2-year all-cause mortality after cancer diagnosis according to quartiles (Q) of plasma miR-145. Model 1: adjusted for age at cancer diagnosis, sex and body mass index at baseline; model 2 adjusted for pack-years and cardiovascular disease in addition; model 3 adjusted for cancer stage and cancer site in addition. PY refers to person-years.

The HRs for 2-year all-cause mortality following cancer diagnosis across quartiles of miR-145 are shown in Figure 4. In general, plasma miR-145 levels were not associated with 2-year mortality after cancer diagnosis in the crude (Model 1 HR Q4 vs. Q1: 1.10, 95% CI: 0.59–2.03) or the fully-adjusted (Model 3 HR 0.99, 95% CI: 0.51–1.91) models.

## 3. Discussion

In this population-based cohort study, we found that participants with the highest expression level of plasma miR-145 had a 44% increased risk of future first-lifetime cancer compared to participants with the lowest expression level of miR-145 in a model adjusted for age, sex, BMI and pack-years of smoking. However, no significant association of plasma miR-145 levels was observed for either the risk of future distant metastatic cancer or for 2-year mortality after cancer diagnosis.

Our finding of an association between high plasma miR-145 and risk of future cancer might seem paradoxical, considering the well-documented tumor-suppressor properties of miR-145 [14]. This perception is based in part on numerous case-control studies that have consistently reported a downregulation of miR-145 in cancerous tissues compared to their normal counterparts [21,22,23]. However, variations in the cell-type composition of cancerous vs. healthy tissue can confound those results [12]. For example, biopsies of a normal colon typically contain higher proportions of miR-145-enriched smooth muscle cells compared to their cancerous counterparts [12]. This risk is minimized in our study because the exposure is the level of miR-145 in plasma, which reflects the resultant of several cell types potentially releasing miR-145 into circulation.

While smoking has been suggested to induce the expression of several miRs [24], the risk of confounding by smoking in our study is minimized. When the study population was restricted to those who had zero pack-years (*n* = 970), the relative risk of cancer for people with the highest miR-145 levels was significantly elevated (HR 1.62). The risk estimates eventually dropped to 1.44 when everyone was included, which could be attributed to never-smokers having a lower baseline risk of cancer. However, the persistence of an increased risk of cancer even as smoking became more pronounced in our population suggests a large degree of independence between smoking and miR-145 levels on the risk of cancer. Moreover, pack-years has been used in a validated clinical risk prediction model for lung cancer based on the same cohort [25], indicating that it is an appropriate measure to capture the risk of smoking on cancer.

Despite the prospective nature of this cohort study, the temporal sequence of exposure and outcome could be reversed in the first year of follow-up, as cancer may be occult. To address this, we re-estimated the relative risk when participants who received their cancer diagnosis within one year of enrollment were excluded. This led to attenuation of the results. Nonetheless, people with the highest expression of miR-145 levels still had an elevated risk of future cancer (HR 1.34), suggesting a minimal risk of reverse-causation on the main findings. The preservation of the temporal sequence might offer insight into the apparent conflict between our results and the suggested functional properties of miR-145.

Identified gene targets of miR-145 still provide a strong basis for the notion that miR-145 possesses, at least partially, tumor-suppressor properties. miR-145 has been shown to directly target the core pluripotency factors OCT4, SOX2, and KLF4, and loss of its function impairs differentiation [26]. According to the cancer stem hypothesis, which posits that tumor growth is driven by a rare subpopulation of cells possessing stem cell-like properties [27], the targeting of these factors sculpts an important role for miR-145 in tumorigenesis. However, this role is complicated by a negative feedback loop between OCT4 and miR-145 [26], where OCT4 can repress the miR-145 promoter [26], and, via endogenous pseudogenes that act as natural miR sponges, reduce miR-145’s ability to inhibit OCT4 [28]. Additionally, other gene targets such as Ras-responsive element-binding protein 1 [29] and c-Myc [15], along with miR-145’s involvement in the regulatory network of the central tumor suppressor p53 [15], point to a multifaceted involvement of miR-145 in cancer biology.

There are several speculated mechanisms that can reconcile a tumor-suppressor role for miR-145 and the finding that high levels of miR-145 are associated with increased risk of near-future cancer. Firstly, the presence of a positive feedback mechanism between miR-145 and early stages of oncogenesis is postulated. Abnormal cells might alter the mRNA/miRNA ratio to favor oncogenes over tumor-suppressor miRs. Several studies have demonstrated how this ratio influences the activity of miRs [17], which could be the result of healthy cells responding by increasing the secretion of tumor-suppressor miRs to reestablish equilibrium [17]. Kosaka et al. demonstrated how secreted tumor-suppressor miRs inhibit cancer cells in a process of cell-competition, using miR-143 as an example [30] (which is in the same cluster as miR-145 and assumed to be co-transcribed with it [31]). Selective blocking of miR cellular uptake has been suggested before [30], and could explain how cancer cells can evade the effects of tumor-suppressors.

Another mechanism is one where tumor-suppressor miRs are sequestrated into exosomes. It has been demonstrated that the sorting of miRs into exosomes is a regulated process [32,33], and not merely reflective of the inner cellular environment. Cancer cells can exploit this mechanism to promote their growth by sequestering tumor-suppressor miRs into exosomes, reducing the tumor-suppressing effects within the cancer cells themselves [33]. For example, metastatic gastric cancer cells have been shown to enrich exosomes with let-7 miRs, another known tumor suppressor, to maintain oncogenesis [34]. We speculate that pre-cancerous cells might similarly secrete exosomes enriched with miR-145, thereby suppressing local tumor-suppressor activity and increasing circulating miR-145 levels.

Thirdly, the so-called dilution effect describes how an abundance of potential mRNA targets (oncogenes) can diminish the regulatory efficiency of tumor-suppressor miRs [35,36,37]. Said differently, when a target mRNA is highly expressed, a greater concentration of miR is required to reach the threshold of biological impact [37,38]. Closer to the onset of cancer, the increased oncogenic expression may spread miR-145’s inhibitory effects thin across multiple targets, reducing its overall efficacy, despite its higher expression.

In this study, we found that the expression of miR-145 in the plasma is highest during the 3 years preceding a cancer diagnosis, which provides a degree of epidemiological support to the speculated mechanisms. However, experimental evidence and validation, specific to miR-145, for each of the previously mentioned mechanisms are needed. Direct comparison with results from case-control studies (that indicate downregulation of miR-145 in cancer tissues and/or plasma at or after cancer diagnosis) is challenging because of the different sample collection times. Therefore, while potentially unexpected, our findings are not necessarily contradictory to those from case-control studies. Unexpected results can often propel further research.

Despite being associated with an increased risk of cancer, plasma miR-145 was not associated with distant metastasis, and, consequently, it was also not associated with short-term mortality after cancer diagnosis. These results provide further evidence against a promoter role for miR-145 in tumorigenesis, contrary to what might be inferred from the elevated cancer risk results. These findings are consistent with research conducted by Akao et al., which included patients with colorectal cancer (*n* = 63) and colorectal adenomas (*n* = 65) [21]. Their study revealed that although miR-145 is downregulated in the early phases of adenoma formation, this modulation does not persist through the progression phase or influence clinical prognostic factors. Interestingly, in the same study, the authors showed a beneficial effect of chemically modified miR-143 for the treatment of colorectal tumors. This emphasizes that while the regulatory machinery of miRs is complex, dependent on temporal and cellular factors and target-interactions, the effort to understand them is justified by the potential benefits in terms of insight into disease mechanisms or as therapeutic targets.

Our study’s strengths include recruiting participants from a large, population-based cohort and the objective validation and completeness of cancer events. The incidence and distribution of cancer sites and stages in our cohort resembled those in a Scandinavian cohort of 144,952 participants, indicating a representative sample [39]. The prospective design with a long follow-up time offers insight into the state of plasma miR-145 prior to the formation of cancer, but the lengthy follow-up time could introduce regression dilution bias. Sensitivity analyses indicated this to a slight degree. Even though the results were attenuated over time, the direction of the risk estimates was preserved with a magnitude consistently greater than 1.3. As genetic determinants of plasma miR-145 levels are still largely unknown, and ethnic variations have been reported [40,41,42,43], our findings predominantly apply to individuals of Caucasian descent. Finally, as in all observational studies, the potential presence of residual confounding cannot be totally ruled out.

The findings of this study should be interpreted within an etiological framework, as they provide novel epidemiological support for the need to account for temporal dynamics when evaluating the impact of tumor-suppressor miR levels on future cancer risk. These findings also challenge the notion that a putative tumor-suppressor would solely be protective against cancer development. As a result, future research with an etiological or hypothesis-generating focus can build on this study to further understand the role of miR-145 in tumorigenesis. On the other hand, although the distribution of cancer types in this study reflected that of the general population, the study was underpowered to assess the correlation between miR-145 and the risk of specific cancer subtypes, thereby limiting its clinical applicability. In this context, future research aiming to evaluate plasma miR-145 levels as a risk biomarker for cancer should focus on specific cancer subtypes, ensuring a well-powered sample size, and including diverse population groups.

## 4. Materials and Methods

### 4.1. Study Population

The Trøndelag Health Study (HUNT) is a population-based cohort study of inhabitants of the (former) Nord-Trøndelag county in Norway. In 2006–2008, all inhabitants ≥ 20 years were invited to participate in the third survey (HUNT3), and 50,807 (54%) individuals attended. All participants provided written informed consent for participation and use of data for medical research.

A subsample of 2000 participants was used to reduce the cost associated with miR analysis. This subsample was originally selected for a case-subcohort study where the outcome was venous thromboembolism, with participants age-matched to the cases [44]. Participants with no available plasma sample or poor plasma quality (*n* = 98) and those with previous cancer (*n* = 162) were excluded, resulting in a final cohort of 1740 participants. Participants were followed from the date of inclusion, and all first-lifetime cancer events occurring during the follow-up period were recorded up until 31 December 2019, yielding a maximum follow-up period of 13.2 years.

### 4.2. Baseline Information

Baseline information for all participants was collected by self-administered questionnaires, physical examination, and plasma samples during inclusion in the study [45,46]. The questionnaires extracted information on smoking habits, and the reported number of cigarettes smoked daily and years of daily smoking were used to calculate pack-years (packs × years, with one pack containing 20 cigarettes). History of cardiovascular disease (CVD) (yes/no) was defined as self-reported previous incidence of myocardial infarction, stroke or angina. BMI was calculated as kg/m^2^ for all participants, as their weight (kg) (wearing light clothing) and height (m) were measured by physical examination.

Blood samples were collected from all participants, and plasma was prepared by centrifugation (3000× *g* for 10 min), frozen and stored at −80 °C in the HUNT Biobank (Levanger, Norway). The plasma samples were retrieved and shipped on dry ice to QIAGEN Genomic Services (Hilden, Germany). Total RNA was extracted with the miRNeasy Plasma Advanced Kit (Qiagen, Hilden, Germany), reverse transcribed with the miRCURY LNA RT Kit (Qiagen, Hilden, Germany), and miR-145-5p expression was measured by qPCR using specific primers and SYBR Green, alongside reference genes and controls for extraction and transcription efficiency. More details are provided in a previous study [44].

### 4.3. Outcomes

Incident cancer diagnoses during follow-up were identified by linkage to the Cancer Registry of Norway using participants’ individual unique national civil registration number. Cancer registration and reporting cancer cases has been mandatory by law since 1951 in Norway, and the registry receives information from several medical sources such as general practitioners, hospital physicians, pathological laboratories and death certificates [47]. The registry has a completeness of 98.8% with a percentage of microscopically confirmed diagnoses of 94%, and it provides information regarding the cancer diagnosis date, cancer localization (ICD-7 codes), histological grade and cancer stage (localized, regional, distant or unknown) [47]. Subjects diagnosed with non-melanoma skin cancers (ICD-7 code 191) and without any other cancer diagnoses were considered cancer-free, owing to the non-metastatic potential of this disease. Table S3 details the definition of general cancer site used in this study based on the ICD-7 codes.

Information on all-cause mortality and migration was obtained by linkage to the National Population Registry of Norway.

### 4.4. Statistical Analysis

The expression level of miR-145 was normalized by that of miR-425-5p using the ΔΔCt method, as the latter has been identified as a suitable housekeeping gene and has been used as a reference gene previously [48,49]. This choice was also supported using both the GeNorm [50] and BestKeeper [51] algorithms on our dataset. The study population was divided into quartiles based on the normalized expression levels of miR-145, with the fourth quartile containing the participants with the highest miR-145 levels. The quartiles were derived for each sex separately, as large differences between the levels of miR-145 have been reported between males and females [52]. Baseline characteristics for all participants and across sex-specific quartiles of miR-145 were presented using mean (±standard deviation [SD]) for continuous variables and percentages for categorical variables.

For survival analysis, follow-up time was calculated from the date of inclusion in the HUNT study (2006–2008) until the earliest occurrence of cancer diagnosis, death, migration or end of follow-up (31 December 2019). Cox proportional hazards regression models were used to estimate hazard ratios (HRs) with 95% confidence intervals (CI) for cancer according to quartiles of miR-145, using the lowest quartile as reference. The proportional hazards assumption was assessed by inspecting the complementary log-log transformation of the survival curves for the miR-145 quartiles. Potential confounding was handled by including age, sex and BMI at baseline as covariates in Model 1, and pack-years as an additional covariate in Model 2.

To assess whether miR-145 was associated with cancer severity, we performed three additional analyses, treating each known cancer stage at diagnosis as a separate outcome: local, regional and distant metastasis. Similarly, we also performed analysis for each general cancer site separately.

To assess whether miR-145 was associated with 2-year mortality post cancer, we used a Cox regression model from the time of cancer diagnosis until either the date of death or two years after cancer diagnosis, whichever came first. In this analysis, only participants who had cancer were included. We applied three models with progressive adjustments: Model 1 for sex, age and BMI at baseline; Model 2 for CVD and pack-years at baseline and Model 3 for the site and stage of cancer at diagnosis.

Five sensitivity analyses were performed for the main analysis of cancer as the outcome. The first one modeled the normalized and scaled expression level of miR-145 as a continuous variable using a smoothing spline with four degrees of freedom to uncover any potential non-linear effect on the risk of cancer. The risk estimates were in reference to a scaled miR-145 value of zero. The second sensitivity analysis addressed the potential of regression dilution bias that could have been introduced due to the long follow-up time [53]. We varied the follow-up time from 1 year up until the maximum follow-up time (13.2 years), while maintaining the same sample size. In the third sensitivity analysis, participants who were diagnosed with cancer within the first year of inclusion in the study were excluded. This analysis was done to minimize the “cart before the horse” risk, as these participants might have had occult cancer at enrollment. A fourth analysis was conducted to assess how miR-145 levels vary with temporal proximity to the cancer event and in comparison with controls who are cancer-free. The follow-up period was divided into 1-year intervals. Within each interval, we compared the average miR-145 levels between individuals diagnosed with cancer and those who remained cancer-free throughout the follow-up period. For each cancer case, we randomly selected five control subjects using density sampling matched by age (within a 5-year range) and sex. A locally estimated scatterplot smoothing (LOESS) technique, weighted by sample size, was used to identify patterns in the mean miR-145 levels for both groups. Finally, analysis which progressively included participants based on increasing values of pack-years (from 0 to the observed maximum of 95 pack-years) was performed to compute risk estimates for participants with similar smoking exposure.

All analyses were undertaken using R version 4.3.2 (The R Foundation for Statistical Computing, Vienna, Austria).

## 5. Conclusions

Our study showed that individuals with the highest expression levels of plasma miR-145 had an increased risk of future first-lifetime cancer compared to those with the lowest expression levels of plasma miR-145. This persisted when accounting for age, sex, BMI and smoking. However, this association did not extend to influence cancer stage at diagnosis or mortality within two years of diagnosis. Further research is needed to validate plasma miR-145 as a potential predictive biomarker of cancer and its contribution to advancing cancer risk stratification.

## Figures and Tables

**Table 1:** Distribution of baseline characteristics for the entire study population and across the four quartiles (Q) of miR-145.

	All	Q1	Q2	Q3	Q4
*n*	1740	436	435	435	434
Age, years	64.9 ± 13.5	64.3 ± 13.8	64.4 ± 12.8	64.2 ± 13.2	66.8 ± 14.0
Sex (female)	55% (955)	55% (239)	55% (239)	55% (239)	55% (238)
BMI, kg/m^2^	27.4 ± 4.3	27.6 ± 4.3	27.8 ± 4.3	27.4 ± 4.4	26.8 ± 4.2
Pack-years	7.6 ± 12.6	8.6 ± 13.5	8.7 ± 13.6	6.8 ± 11.8	6.4 ± 11.2
CVD *	15% (263)	16% (68)	13% (58)	16% (71)	15% (66)
Diabetes *	7% (125)	7% (29)	8% (33)	9% (40)	5% (23)

BMI indicates body mass index; CVD, cardiovascular disease (history of myocardial infarction, stroke, angina pectoris). * Self-reported at baseline.

**Table 2:** Characteristics of participants at cancer diagnosis and distribution of cancers across stages and general site (*n* = 300).

	Values
Years to cancer	6.4 ± 3.5
Age at cancer, years	76.0 ± 10.2
Sex (female)	42% (126)
**Stage**	
Local	35% (105)
Regional metastasis	29% (86)
Distant metastasis	19% (57)
Unknown	17% (52)
**General site**	
GI *	25% (76)
Prostate	19% (58)
Lung	9% (28)
Hemo/lymph	9% (27)
Urological	8% (24)
Breast	7% (22)
Malignant melanoma	6% (18)
HPB **	6% (17)
Gynecological	5% (15)
Others	5% (15)

* GI indicates Gastrointestinal tract and includes upper GI and colorectal cancers. ** HPB indicates hepato-pancreato-biliary cancers and includes cancers of the liver, pancreas and bile ducts.

## Data Availability

Access to data from the HUNT study can be obtained by application to the HUNT administration (https://www.ntnu.edu/hunt/data (accessed on 30 November 2024)).

## Supplementary Materials

The following supporting information can be downloaded at: https://www.mdpi.com/article/10.3390/ijms26052191/s1.

## Abbreviations

ΔΔCt	Delta-delta threshold cycle
BMI	Body mass index
CI	Confidence interval
c-Myc	Cellular myelocytomatosis oncogene
CVD	Cardiovascular disease
GI	Gastrointestinal tract
HPB	Hepato-pancreato-biliary
HR	Hazard ratio
HUNT	The Trøndelag Health Study
ICD	International Classification of Diseases
KLF4	Krüppel-like factor 4
LOESS	Locally estimated scatterplot smoothing
miR	MicroRNA
miR-145	MicroRNA-145-5p
OCT4	Octamer-binding transcription factor 4
p53	Tumor protein 53
PCR	Polymerase chain reaction
qPCR	Quantitative real-time PCR
Q	Quartile
RT-qPCR	Reverse transcription quantitative real-time PCR
SD	Standard deviation
SOX2	Sex determining region Y-box 2
