# Supplementary material for: Plasma miR-145-5p Levels and Risk of Future Cancer—Results from the HUNT Study
Source: Int J Mol Sci. 2025 Feb 28;26(5):2191. doi: 10.3390/ijms26052191 (PMC11899732; doi:10.3390/ijms26052191)
Supplement: Supplementary file 1 [file ijms-26-02191-s001.zip › ijms-3500659-supplementary.pdf]

Supplementary Figures

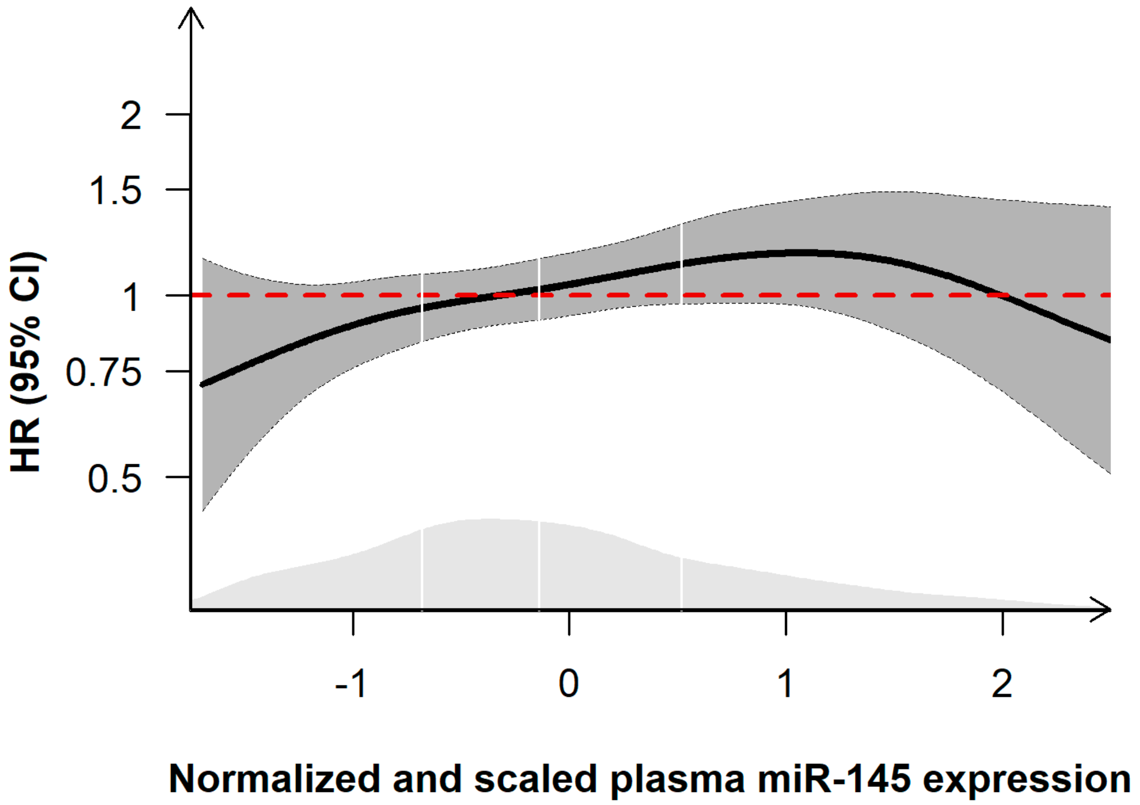

Figure S1. Estimated cancer risk of cancer as a function of miR-145 plasma expression levels modelled as a spline. This Cox regression model was adjusted for sex, age, body mass index and pack-years at baseline, and the primary exposure was the normalized and scaled miR-145 levels entered as a smoothing spline with four degrees of freedom. The solid lines depict hazard ratios (HR) relative to scaled expression of zero. The red dashed line depicts a hazard ratio of 1.00. The surrounding shaded area corresponds to the 95% confidence interval (CI) around the estimated risk. The distribution of scaled miR-145 plasma levels is shown at the bottom of the plot, and the vertical white lines correspond to the 25th, 50th and 75th percentile of miR-145 levels.

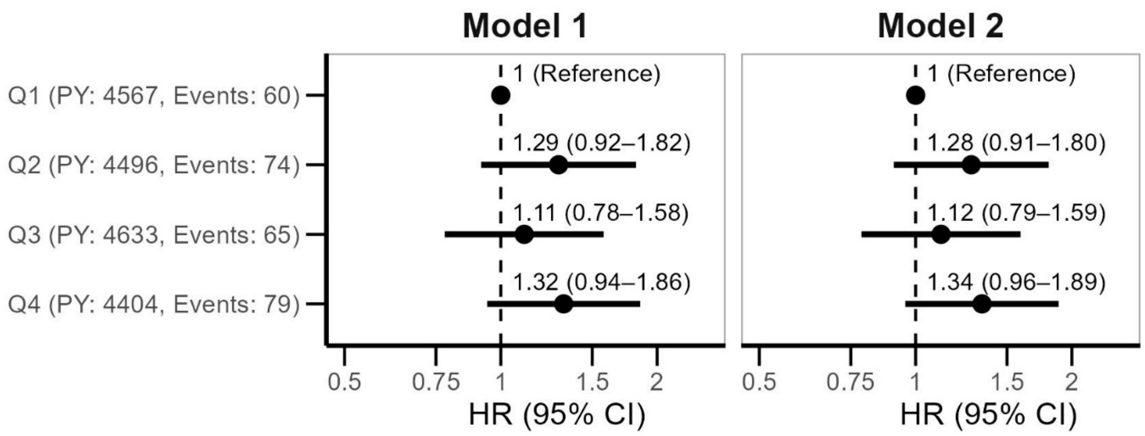

Figure S2. Hazard ratios (HRs) with 95% confidence intervals (CIs) of overall cancer according to quartiles of plasma miR-145 excluding participants who got their cancer diagnosis within one year of enrollment. Model 1: adjusted for age, sex, and body mass index at baseline; Model 2 adjusted for age, sex, body mass index, and smoking pack-years at baseline. PY refers to person-years.

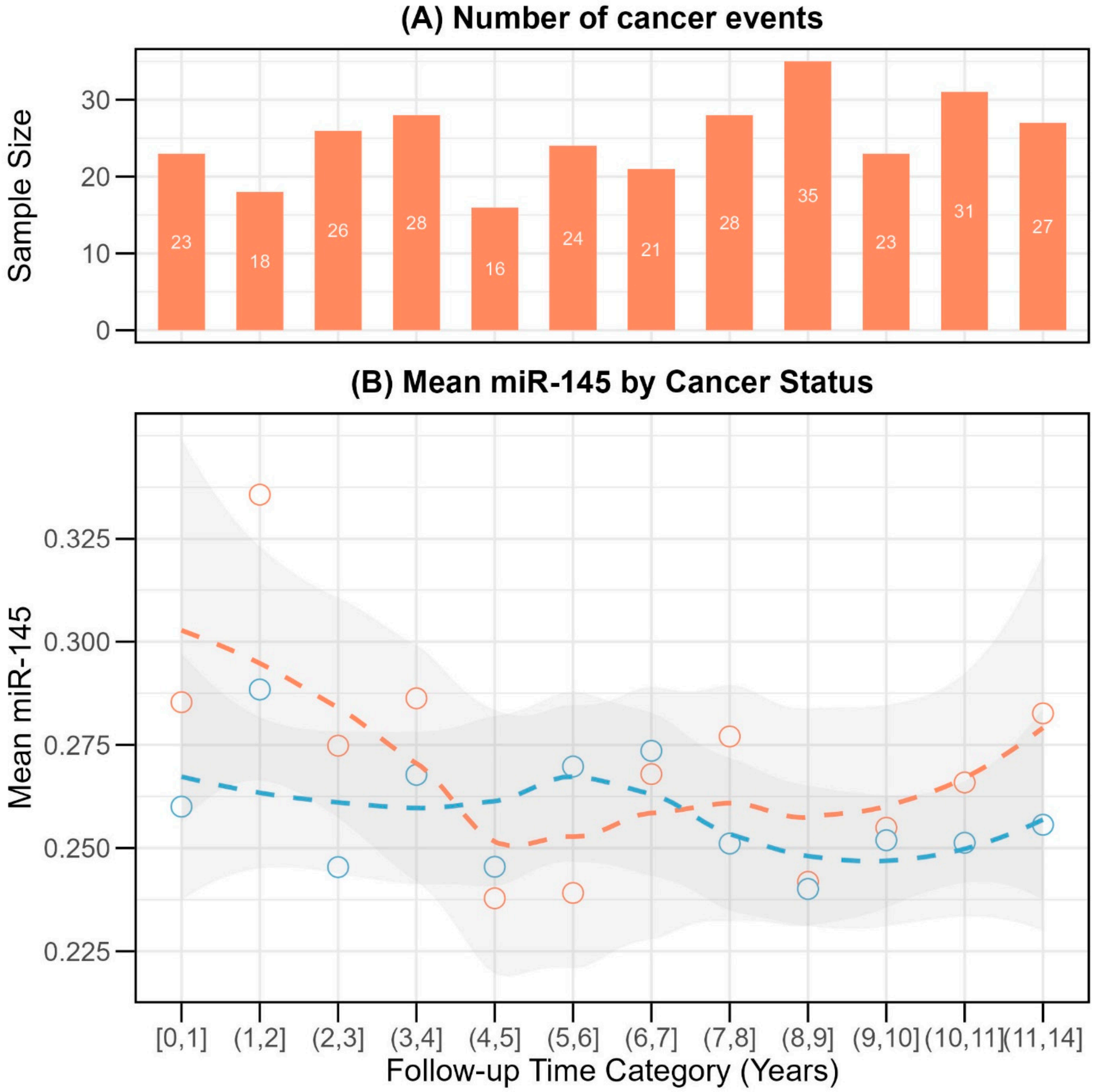

Figure S3. Panel (A) depicts the number of cancer events in each follow-up period. Panel (B) shows the mean miR-145 levels for the cases (in orange) and matched controls (in blue) over varying follow-up times. The dashed line represents the weighted smoothing line, and the gray shaded area indicates the confidence interval around this estimate.

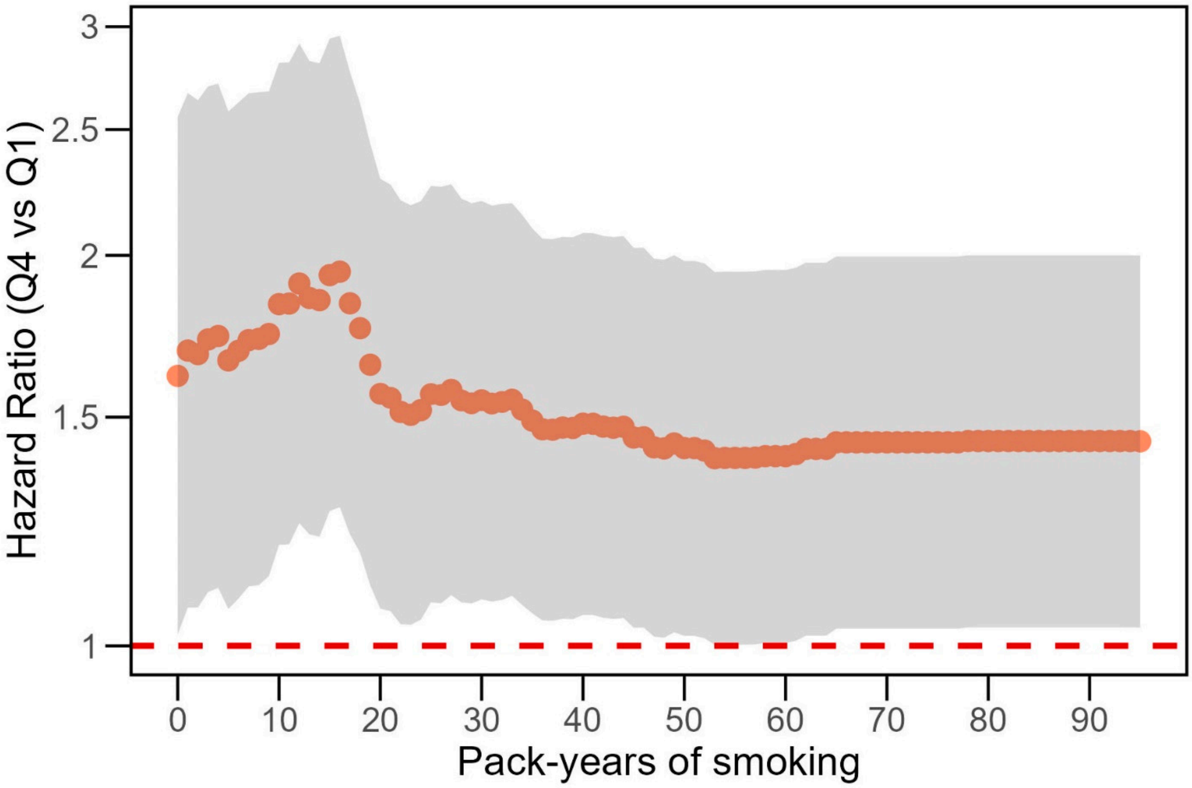

Figure S4. Estimated hazard ratios (HRs) with 95% confidence intervals (CIs) of cancer as a function of smoking pack-years from 0 to the observed maximum of 95 pack-years. Analysis was adjusted for sex, age and body mass index at baseline. Individuals with miR-145 level in the highest quartile (Q4) were compared with those in the lowest quartile (Q1, reference). Orange dots indicate the hazard ratio. Dashed horizontal line corresponds to a HR of 1.00, while the shaded gray area corresponds to the 95% CI around the risk estimates. All estimates have a corresponding *p-value* < 0.05.

Supplementary Tables

Table S1. Definition and distribution of the cancers classified as "Others".

**Others**	***n***
Central nervous system	4
Thyroid gland	4
Glottis	2
Nasal cavity	1
Parotid gland	1
Unknown	3

Table S2. Hazard ratios (HRs) with 95% confidence intervals (CIs) of each general cancer site according to the highest quartile (Q) of plasma miR-145. The model was adjusted for sex, age, body mass index and pack-years at baseline.

General cancer site	n	Events	HR (95% CI)
Gastrointestinal tract	1740	76	1.75 (0.84, 3.66)
Prostate	785	58	0.99 (0.47, 2.10)
Lung	1740	28	4.24 (1.18, 15.23)
Hemo/lymph	1740	27	0.78 (0.26, 2.32)
Urological	1740	24	1.21 (0.40, 3.63)
Breast	955	22	0.75 (0.26, 2.16)
Malignant melanoma	1740	18	3.35 (0.91, 12.28)
Hepato-Pancreato-Biliary	1740	17	0.80 (0.18, 3.62)
Gynecological	955	15	0.80 (0.18, 3.60)
Others	1740	15	7.33 (0.87, 61.36)

Table S3. Definition and groupings of general cancer sites based on ICD-7 code ranges.

ICD range	General cancer site
1530-1549	Gastrointestinal tract (Colorectal)
1500-1529	Gastrointestinal tract (Upper GI)
1993	Gastrointestinal tract (GI)
1570-1579	Hepato-Pancreato-Biliary (HPB) (Pancreatic)
1550	Hepato-Pancreato-Biliary (HPB) (Liver)
1551	Hepato-Pancreato-Biliary (HPB) (Intrahepatic bile ducts)
1620-1699	Lung
1700-1709	Breast
1710-1769	Gynecological
1770-1779	Prostate
1780-1899	Urological
2000-2079	Hemo/lymph
1900-1909	Malignant melanoma
Others	Others
